# Homœopathy *vs.* Allopathy

**Published:** 1897-02

**Authors:** George J. Augur

**Affiliations:** Oakland, Cal.


### HOMŒOPATHY *versus* ALLOPATHY.

GEO. J. AUGUR, M. D., OAKLAND, CAL.

I use the term allopathy, though recognizing the fact that those of that school reject the appellation as a term of opprobrium, because given to them by Hahnemann, rather than for the reason that they do not recognize in its definition the true explanation of their theory.

The *Century Dictionary*, prepared under the superintendence of Prof. William Dwight Whitney, of Yale, defines allopathy in medicine as a therapeutic method characterized by the use of agents producing effects different from the symptoms of the disease treated—while in smaller type it states, "The name is incorrectly applied in distinction from Homœopathy to the traditional school of medicine, which opposes the homœopathic theory." Notwithstanding this high authority, I recognize the term as just and comprehensive as applied in the main to that school. In support of their position of the incorrectness of the term allopathy, there is frequently cited the action of emetics in some kinds of indigestion and Rhubarb in some kinds of diarrhœa, tending to show that the old-school practice is not confined to the limited sphere of prescribing drugs which in their action produce effects contrary to those noticeable in the disease for which the drug is administered. While I admit that every well-informed allopathic physician knows well that Ipecac and Rhubarb have a double action, what allopathic physician gives Ipecac alone to cure vomiting, or Rhubarb alone in diarrhœa, uncombined with astringents or opiates? The nearest approach to this is made by Dr. J. Lewis Smith, an established authority on diseases of children, when in certain conditions of diarrhœa he recommends a prescription composed of Magnesia-sulph., Tinct. Rhu., Syr. Zingiberis and Aqua-carui, but immediately following this he says: "The effect of laxative medicine employed for the purpose of correcting the functions of the gastro-intestinal surface is uncertain, we must rely on astringents and opiates. Much harm is often done and precious time lost by prescribing laxative mixtures when opiates and astringents are required." I think this illustration proves the correctness of the term allopathy, though used by them to prove the contrary.

It has been claimed that the homœopath in the treatment of disease confines himself to the amelioration of symptoms, while the allopathic physician, more scientific (?) in his method, applies his remedies to the morbid condition constituting the disease.

With all due respect to my former colleagues, many of whom are honestly ignorant, while more are dishonorably prejudiced; it would be more proper, nay truthful, to reverse the statement and then apply it to the two schools; for if our remedies do not reach the morbidly-altered vitality represented by symptoms according to the law of similars, rather than one or more symptoms of this altered state, who will explain why, when the proper remedy has been selected, one symptom after another, amounting in many instances to twenty or more, disappear, until perfect health is restored? How can perfect health be restored if the disease is not reached through symptoms? We deal with symptoms, it is true, but only as signs representing a pathological condition, which taken collectively enable the conscientious follower of the true healing art to select the drug which will produce a condition of ill-health closely allied in symptoms to the disease he is called to treat.

If we homœopathic physicians treat symptoms and not the primary cause of the symptoms, and our remedies are simply an apology for doing some remedial good, while nature does the work, will some of those wise gentlemen tell me why and how, in some cases of dysmenorrhœa when the suffering is most intense, immediate relief is given; or how, in a case of amblyopia or amaurosis due to spinal irritation accompanied by photophobia and lachrymation, with paralysis of some of the orbital muscles, and intense cerebral pain, and muscular tremor of the arms; or how, in a case of vesical neuralgia, after months of suffering in spite of the best allopathic skill, immediate relief was given followed by ultimate cure: The treatment being the single remedy and high potency?

I might cite numerous other cases as having fallen to my lot to treat, but the above is sufficient to illustrate my point, that Homœopathy when practiced according to the teachings of Hahnemann is more scientific, and more capable of reaching the true pathological condition, and restoring the damaged organ to the normal condition constituting perfect health than the so-called rational treatment, a name incorrectly applied to our opponents. In seeking to search for and remove the cause, upon which claim the appellation "rational" is made, they, it is true have in their ranks many scientific investigators; but do they alone comprise the scientific world as applied to medicine in microscopical and pathological research? I think not.

Koch's tuberculin, about which great discovery the so-called regular profession at one time made such a great ado, now is scarcely mentioned by them, though the homœopaths, it is authoritatively stated, employed potentized tubercular bacilli years before Koch was heard of, and continue to do so now. The same may be said of Pasteur's great discovery for the cure of hydrophobia by inoculating with the poison of the mad dog. What one among those of the old school who has gained repute for medical disclosures used by them can compare with Hahnemann in the greatness of his discoveries; in the originality of the principle involved and its scientific application? What one of them can compare with him as the excogitator of a system of medicine, or even of a therapeutic measure discovered by means of the microscope, or in the search for pathological germs, or in the field of chemical analysis? A system which, on the one hand, has caused so much comment, so much adverse criticism, so much united effort to crush; while on the other it has brought to its support from among those who once denounced it more ardent supporters, both from the medical profession and educated laity, and has ameliorated more suffering without physical and mental perturbation, and which has been a greater boon to humanity in the prolongation of life. As for their treatment being rational, is it rational, say nothing of scientific, to give such large doses of dangerous drugs to jeopardize the patient's life?

Is it in accord with good reason, in cases of continued fever, no matter what the cause may be, to give Phenacetine or some other coal-tar derivative, in such doses as to produce cyanosis? Is it right to give Aconite to a child in doses which threaten collapse? Look at the effect of Aconite, or *any* nerve or arterial sedative in allopathic doses given for its antipyretic effect! Does it so act upon the diseased condition as to favor restoration to health? Can any remedy of this kind be right that is given in doses sufficiently large to cause cyanosis, a condition of imperfect circulation and oxygenation of the blood? Can any sedative given in doses to produce its physiological effect, and thereby lower an abnormal temperature several degrees, be of therapeutic advantage? In just the proportion that the temperature is rapidly lowered by the physiological action of a drug, in just that degree must the nervous system be depressed, and in proportion to the nervous depression must the vital powers be incapacitated to throw off the disease, and the resisting power ever after lessened. In support of this position let us review the action of sedative medicines, according to a leading allopathic authority, which says: "Sedatives are medicines which depress nervous force. Some affect nervous force in general; others confine their action to particular nerves. They are mostly energetic and dangerous agents. For the time being they destroy nervous power, and remove nervous control." Let us see the effect of a special sedative on the vagus nerve. It would probably by its action on the rhythmical action of the heart cause it to beat abnormally slow and irregular. It would be likely by its action on the lungs to diminish the desire for breath, and lessen the irritability of the pulmonary mucous surfaces. I presume it was from reasoning in this way that a man of deep thought, one of the visiting physicians at one of the leading allopathic hospitals in the East, during my term of service there, gave it as his opinion that sedatives in pneumonia given with the view of reducing temperature, were absolutely harmful, and to my personal knowledge, rather than subject the patient to the usual risks attending the old school mode of treatment, he treated a severe case of this disease with simply colored water.

What physician who is familiar with the lasting benefits derived from infinitesimal doses of medicine administered according to the law of similars, and who consequently must believe in their dynamic force, is prepared to doubt the permanent injury to nerve centres by derangement of their molecular construction? A fall upon the head can entail serious consequences by simply producing a commotion in the brain-substance, without any organic change as pathologists are led to infer being recognizable. In such cases, according to Erichsen, a permanently irritable state of the brain may be left; the patient yet capable of the ordinary duties of life, but becoming readily excited, though not to an inordinate intensity, by slight excesses in diet or by the use of stimulants or by mental emotion. If, therefore, permanent injury to the brain can follow without a localized organic change being discoverable, is it less reasonable to suppose that drugs like Antipyrine, Acetanilide, and other medicines of a kindred nature, given in repeatedly large doses, so that the effect of the drug is noticeable first by the functional change in the circulatory and respiratory organs, which is primarily brought about by derangement of the nervous system, can and do produce a molecular change in the nerve centres which is injurious and lasting in its effects? Shall we claim that because the drug falls short of producing death by its physiological action, therefore no lasting trace of its effect remains, because, forsooth, it is not recognizable on the surface? Such conclusions, it seems to me, would be illogical and devoid of deductive reasoning.

Aside from the permanent injury of the nerve centres, there are other phenomena dependent upon the generating and controlling force of these centres and their branches which act as carriers, by virtue of which centres and their capacity to generate healthful and inhibitory impressions depends the normal and physiological action of every organ in the body. If the impression given off from a nerve centre is abnormal then must the nutritive changes in the organs to which its branches are distributed be abnormal. Let the lungs, heart, liver, kidneys, and alimentary canal become changed in the performance of their functions from the normal and what would be the consequence? Take the alimentary canal, for instance, not only does it preside over the function of digestion in its peculiar way, transforming albuminoid substances into peptones, but also gives birth to "alkaloidal poisons," and toxic substances resulting from intestinal putrefactions. While the stools eliminate the greater part of these poisons, which are expelled with them, the intestinal mucous surface absorbs a part, which in turn is taken up by the blood and eliminated by the kidneys. Now, let the nerve-supply to this canal alone become deranged, if it were possible, and the result would probably be manifest first in the changed condition of the digestive secretion, while this in turn would be followed by defective digestion, increasing in consequence the amount of alkaloidal poisons normally present, and decreasing in quantity the assimilable properties, as a result the percentage of toxic principle in the blood would be augmented beyond the power of the excretory organs to throw off, and this in turn would react upon the nervous system, entailing various systemic derangements. Recognizing, as we must, the nervous system as an "intermediary" in the production of disease, and disturbances of nutrition as the causative agent in a large number of chronic diseases, what is more rational, if we wish to maintain a high standard of health, to preserve without injury the intermediary link—the nerves—that the process of vital cell-life—comprising nutrition—may not become permanently deranged. To avoid the one and preserve the other Homœopathy will do more than any other system of therapeutics has done or can do.

